# Methylome Characterization of Burkholderia pseudomallei Strain 982 at Single-Base Resolution

**DOI:** 10.1128/MRA.00898-19

**Published:** 2019-10-24

**Authors:** Kar-Wai Hong, Kok Keng Tee, Wai-Fong Yin, Richard J. Roberts, Kok-Gan Chan

**Affiliations:** a International Genome Centre, Jiangsu University, Zhenjiang, China; b Department of Medical Microbiology, Faculty of Medicine, University of Malaya, Kuala Lumpur, Malaysia; c New England Biolabs, Ipswich, Massachusetts, USA; d Division of Genetics and Molecular Biology, Institute of Biological Sciences, University of Malaya, Kuala Lumpur, Malaysia; University of Southern California

## Abstract

Burkholderia pseudomallei is the etiological agent of melioidosis, which has been studied by transcriptome and secretome analyses. However, little is known about the methylome of this pathogen. Here, we present the complete genome and methylome of melioidosis-causing B. pseudomallei strain 982.

## ANNOUNCEMENT

Burkholderia pseudomallei is a Gram-negative bacterium from the class *Betaproteobacteria* that causes melioidosis with a case fatality rate of 10 to 50% (1, 2).

Here, we have sequenced B. pseudomallei strain 982 in order to understand its methylome. This strain was originally isolated from the pus of a 13-year-old male patient on 21 March 2015 in Pahang, Malaysia. The pus sample was serially diluted prior to being spread on blood agar followed by incubation at 37°C to obtain a single colony (3). The bacterial sample was identified using the analytical profile index (API) approach and a Microflex benchtop matrix-assisted laser desorption ionization–time of flight (MALDI-TOF) mass spectrometer (Bruker Daltonik GmbH, Germany) (4). This isolate was cultivated in lysogeny broth, and its genomic DNA was extracted using a MasterPure DNA purification kit (Epicentre, Madison, WI, USA). The quantity and quality of the extracted genomic DNA were measured using a Qubit 2.0 fluorometer and a NanoDrop 2000 spectrophotometer (both Thermo Fisher Scientific, Waltham, MA, USA) and a Pippin Pulse electrophoresis power supply (Sage Science, Beverly, MA, USA), respectively (5). Genomic DNA (10 μg) was sheared using a g-TUBE (Covaris, Woburn, Massachusetts, USA). A 20-kb template library was prepared using the BluePippin size-selection system and sequenced using a Pacific Biosciences RS II platform in two runs of the same batch by using P5-C3 chemistry following the MagBead loading protocol (all Pacific Biosciences, Menlo Park, CA) (6, 7), as this increases sequencing coverage, which will facilitate downstream analysis.

*De novo* assembly of the genome was performed using the Hierarchical Genome Assembly Process (HGAP) version 2.0 (8). The circularity of the assembled genome was studied using Gepard (9) and Contiguity (10). The overlapping ends were trimmed using Minimus2 of the AMOS software package (11). The annotation and detection of prophages were performed using the NCBI Prokaryotic Genome Annotation Pipeline (PGAP) and the PHAge Search Tool Enhanced Release (PHASTER) (12, 13). For base modification analysis, the RS_Modification_and_Motif_Analysis.1 protocol (https://github.com/sanger-pathogens/Bio-PacbioMethylation/blob/master/lib/Bio/PacbioMethylation/RSModificationRunner.pm) with a default modification quality value score of 30 was used, and the motif analysis summary was deposited in REBASE (14). Default parameters were used for all software unless otherwise specified.

A total of 527,043,402 reads and 458,304,859 reads were generated from two individual SMRT Cell v2 instances with *N*_50_ values of 9,087 bp and 9,162 bp, respectively, giving an average sequencing coverage of 116.91×. This bacterium consisted of two circular chromosomes of 4,028,032 bp (67.87% G+C content) and 3,156,645 bp (68.56% G+C content). It contained 6,199 genes, 6,035 of which are protein-coding genes and 61 of which are tRNA genes, with 4 rRNA operons (5S, 16S, and 23S) and 1 noncoding RNA. PHASTER indicated that chromosome 1 harbored one intact prophage region, while chromosome 2 possessed two incomplete prophage regions.

Four methylation motifs were detected, namely, GTC^m6^ANNNNNNTGG, GTWW^m6^AC, CAG^m6^ATG, and CAC^m6^AG (Table 1). By performing a comprehensive search in REBASE, the DNA methylase (MTase) which is responsible for the motif GTWWAC was found to be conserved across the *Burkholderiaceae* family (14, 15).

**TABLE 1:** MTase and its predicted DNA recognition sequences in B. pseudomallei strain 982

RM system gene annotated	Chromosome	Type (subtype)	Locus tag (start bp–stop bp)	Predicted recognition motif^*a*^	Methylated type
M.Bps982II	1	I (gamma)	AMS56_03820 (799640–801286)	GTC^m6^**A**NNNNNNTGG	^m6^A
S.Bps982II	1	I	AMS56_03825 (801276–802589)	GTCANNNNNNTGG	
Bps982IIP	1	I	AMS56_03830 (802586–805717)	GTCANNNNNNTGG	
M.Bps982ORF1205P	1	II (beta)	AMS56_01205 (247616–248392)^*b*^		
M.Bps982ORF17150P	1	II	AMS56_17150 (3830654–3831883)		
M.Bps982I	1	III (beta)	AMS56_01845 (377543–379576)^*b*^	CAC^m6^**A**G	^m6^A
Bps982IP	1	III	AMS56_01840 (374473–377502)^*b*^	CACAG	
M.Bps982IV	1	III (beta)	AMS56_02205 (546377–547897)	CAG^m6^**A**TG	
Bps982IVP	1	III	AMS56_02210 (547907–550591)	CAGATG	
M.Bps982III	2	II (beta)	AMS56_(1628287–1629135)	GTWW^m6^**A**C	^m6^A
M.Bps982ORF25235P	2	II	AMS56_(1703198–1703761)		
M.Bps982ORF25241P	2	II	AMS56_(1705313–1706002)^*b*^	YGG^m5^**C**CR	^m5^C

a Modified bases are highlighted in bold and underlined when on the complementary strand.

b A complementary strand sequence.

### Data availability.

The whole-genome sequence of B. pseudomallei strain 982 has been deposited at DDBJ/EMBL/GenBank under the accession numbers CP012576 and CP012577 for chromosomes 1 and 2, respectively. The version described in this paper is available under NCBI BioProject number PRJNA293915 and NCBI BioSample number SAMN04011951. The raw reads of sequenced genomic DNA of this bacterium and the output of base modification and motif analysis were deposited in the SRA under accession numbers SRX6717522 and SRZ189982, respectively. The description of genes related to the restriction modification (RM) system of this strain is available in REBASE under the organism number 16827 (http://rebase.neb.com/cgi-bin/onumget?16827).
